# Clinical, haematological and biochemical responses of sheep undergoing autologous blood transfusion

**DOI:** 10.1186/1746-6148-8-61

**Published:** 2012-05-20

**Authors:** Rejane Santos Sousa, Dowglish Ferreira Chaves, Raimundo Alves Barrêto-Júnior, Isadora Karolina Freitas Sousa, Herbert Sousa Soares, Isabella Oliveira Barros, Antonio Humberto Hamad Minervino, Enrico Lippi Ortolani

**Affiliations:** 1 Federal Rural University of Semi-Arid, Av. Francisco Mota, 572, Bairro Costa e Silva, CEP: 59.625-900, Mossoró, RN, Brazil; 2 Department of Clinical Sciences, College of Veterinary Medicine, University of São Paulo, Av. Prof. Dr. Orlando Marques de Paiva, 87 - Cidade Universitária, CEP 05508 270, São Paulo, SP, Brazil

## Abstract

**Background:**

This study aimed to evaluate the clinical, haematological and biochemical responses to autologous blood transfusion and the feasibility of this practice in sheep. Thus, we used eight male, 8 months old sheep, weighing on average 30 kg, from which 15 mL/kg of whole blood was collected and stored in CPDA-1 bags. Blood samples were refrigerated for 8 days and subsequently re-infused. The clinical, haematological and biochemical parameters were evaluated before blood collection and reinfusion, after 10 minutes of collection and reinfusion, after 3, 6, 12, 24, 48, 96 and 192 hours after collection and reinfusion.

**Results:**

With respect to clinical parameters, we observed a decrease in heart rate after 24, 48 and 196 hours from reinfusion compared to basal values (*p* < 0.05). Haematological variables including globular volume and erythrocyte counts showed a significant decrease (*p* < 0.01) at all time points after collection and increased (*p* < 0.01) at all time points after reinfusion. There was a significant increase in total protein and calcium at all time points after reinfusion (*p* < 0.05).

**Conclusion:**

Autologous transfusion in sheep slightly altered the physiological, biochemical and haematological responses of sheep, indicating that the technique proposed is safe and can be applied in the clinical practice of this species. The 8 d period was not sufficient for complete recovery of the haematological parameters after blood collection.

## Background

In recent decades, there has been a considerable increase in the availability and use of homologous blood in veterinary haemotherapy as a result of the advancement of transfusion therapy and optimisation of blood banks [1]. Homologous transfusion is used to treat various diseases and surgical procedures; however, there are several complications associated with administration of blood and its components [2].

The risks of blood transfusions can be reduced by the use of autologous transfusion, or autotransfusion, which consists of pre-donation of a patient’s blood that will later be reinfused into the same patient [3]. The use of autologous transfusion has the advantage of eliminating the risk of immune-mediated haemolytic reactions, virus and prion transmission, febrile reactions, and allergic reactions and reduces the risk of postoperative infection and incidence of cancer that can occur in homologous transfusions due to immune system modulation [4,5].

Although autologous transfusion has been practiced for more than 100 years, there has been an explosive growth of its use in human medicine in recent decades. The reasons for this include economic benefits, greater clinical safety (due to the concern with risks of transfusion-transmitted diseases) and a growing interest in alternatives to homologous transfusion [6,7]. In veterinary medicine, this practice is not widespread; in spite of the use of haemotherapy for the treatment of various veterinary diseases, research to demonstrate the feasibility of this technique in several animal species is necessary.

Because sheep is considered a farm animal, some therapeutic procedures are not economically justified; however, improvement in a national herd is a reality with the introduction of animals of high genetic value. These animals need differential treatment, such as elective surgical procedures that lead to a loss of blood, for which autologous transfusion is the best fit.

Considering the lack of studies related to the use of autologous blood transfusions in sheep, as well as the possible clinical, haematological and biochemical changes caused by this therapeutic practice, coupled with the possible contribution of the technique in veterinary medicine surgical interventions, this study aimed to evaluate the clinical, haematological and biochemical responses of sheep undergoing autologous blood transfusions. We also aimed to verify the feasibility of this technique in sheep.

## Methods

### Animals

This study was performed in accordance with adequate ethical standards and animal care and was approved by the Bioethics Committee of the Institute of Biodiversity and forest from the Federal University of Pará Western, Santarém, Brazil. The study used 8 male, 8 months old sheep, weighing 30 kg on average, that were considered healthy after a physical examination, complete blood count and stool examination. The animals were kept in collective pens. They were dewormed and underwent 30 days of adaptation, during which they were fed roughage concentrate supplemented with mineral salt. After this period, blood samples were collected for complete blood count. Study animals with a globular volume greater than or equal to 30% were used in this study.

### Experimental time points

Blood was collected from sheep, stored, and reinfusion was performed after a period of eight days. We evaluated the clinical, biochemical and haematological parameters before and after collection and reinfusion of whole blood at predetermined intervals: Tc0 (before blood collection), Tc (10 minutes after collection), Tc3, Tc6, Tc12, Tc24, Tc48, Tc96 (3, 6, 12, 24, 48 and 96 hours after collection, respectively), Tc8d (8 days after collection), Tr0 (before blood reinfusion), Tr (10 minutes after reinfusion), Tr3, Tr6, TR12, Tr24, Tr48, Tr96 (3, 6, 12, 24, 48 and 96 hours after reinfusion, respectively) and Tr8d (8 days after reinfusion).

### Preparation of donors and blood collection

The sheep were subjected to withdrawal of 15 mL/kg of whole blood, which amounted to approximately 450 mL. The volume of whole blood collected was the recommend for large animals [8,9]. Trichotomy and asepsis of the jugular region was performed for the blood collection. The animal was subsequently positioned laterally for the jugular venipuncture, and the needle placed in the caudocranial direction. The blood was stored in sterile bags containing citrate phosphate dextrose and adenine solution (Terumo Medical do Brazil) and kept under refrigeration between 3 and 6°C for 8 days. During the storage period, the bags were daily manually inverted to mix the whole blood with the preservative solution.

After the storage period, the blood bags were removed from the refrigerator and kept at room temperature for 30 minutes before reinfusion. The bag was attached to a catheter for administration of the blood (Hemoflex, Equiflex Industry and Trade of Disposable Products LTD), and the blood was reinfused through the jugular vein.

### Clinical, haematological and biochemical parameters

The clinical parameters evaluated at the pre-determined time points included heart rate (HR), respiratory rate (RR), rectal temperature (RT), capillary refill time (CRT) and systolic blood pressure (SBP).

For haematological evaluation, whole blood samples collected through a vacuum system (Vacutainer®) in tubes containing ethylenediaminetetraacetic acid (EDTA) were used to determine the globular volume (GV) by the microhematocrit technique and erythrocyte and leukocyte count (RBC and WBC, respectively) by microdilution in a Neubauer chamber.

Serum samples were used for the biochemical analysis, which were analysed using an automatic biochemical analyser (Labway 2000, Labtest®, Lagoa Santa, MG, Brazil). The total serum protein (TP) concentration was determined by the biuret method, and albumin concentration was determined using the bromocresol green method. Enzymatic activity of Gamma glutamyltransferase (GGT), aspartate aminotransferase (AST) and creatine kinase (CK) was determined using commercial kits (BioSystem S.A., Barcelona, Spain). The enzyme activities were determined at 30°C, following the recommendation of Kaneko et al. [10].

The serum creatinine was determined by the kinetic method using commercial kit (Sigma-Aldrich®, St. Louis, MO, EUA). Serum urea was determined using commercial kit (Sigma-Aldrich®, St. Louis, MO, EUA). The concentrations of calcium (Ca) and phosphorus (P) were determined using commercial kits (BioSystem S.A., Barcelona, Spain).

### Statistical analysis

Statistical analysis was performed using the Minitab statistical software. The paired *T* test was used for comparison of Tc0 and Tr0 with the other time points of collection and reinfusion, respectively. The minimum level of significance adopted was 5%.

## Results

### Clinical evaluation

The collection and reinfusion of 15 mL/kg of whole blood promoted irrelevant clinical alterations in the sheep studied. The Table 1 present data from clinical examination in all time points evaluated. Compared with Tc0, the HR remained unchanged (*p* > 0.05) during blood sampling, although it was was slightly altered due to the stress of restraint. During reinfusion, the HR decreased (*p* < 0.05) at Tr24, Tr48 and Tr8d.

**Table 1:** Means of heart rate (HR), rectal temperature (RT) and capillary refill time (CRT) observed during the experimental time points of blood collection and reinfusion

**Time point**	**HR (Bat/min)**	**RT (°C)**	**CRT (seconds)**	**Time point**	**HR (Beat/min)**	**RT (°C)**	**CRT (seconds)**
Tc0	91	39.4	1.7	Tr0	89	39.3	1.6
Tc	88	*39.0	1.9	Tr	84	39.3	1.8
T3	98	*39.1	1.8	Tr3	84	39.2	1.7
T6	96	39.2	1.9	Tr6	91	39.3	2.1
T12	100	*39.0	1.7	Tr12	87	39.1	2.0
T24	90	*38.9	1.8	Tr24	*78	38.9	2.0
T48	96	*38.8	1.9	Tr48	*76	*38.5	1.8
T96	95	*39.0	2.1	Tr96	82	39.0	1.9
T8d	87	39.2	1.8	Tr8d	*73	38.7	1.9

* Significative difference from time zero (T0 or Tr) throughout paired *T* test (*p* < 0.05).

RT decreased after blood collection at Tc, T3, T12, T24, T48 and T96 but remained within the reference values for the species. There was no difference in CRT (Table 1), RR and SBP (data not shown) between time points (*p* > 0.05).

### Haematological evaluation

GV and RBC (Table 2) showed a decrease (*p* < 0.01) at all time points after blood collection compared to baseline. The blood collection caused a decrease in GV, but these remained within normal limits for the species (27–45%) [11]. GV and erythrocyte counts on Tc until T12 showed a decreased compared to T0, but we noticed a sharper drop on T24. However, from T48 on, there was an increase in both GV and RBC. In contrast, GV and RBC after reinfusion of blood increased (*p* < 0.01) after Tr0, and an increase in GV of about 10% was observed.

**Table 2:** Means of globular volume (GV), red blood cell count (RBC) and white blood cell count (WBC) observed during the experimental time points of blood collection and reinfusion

**Time point**	**GV (%)**	**RBC (x10**^**6**^/**μl**)	**WBC (x10**^**9**^/**l**)	**Time point**	**GV (%)**	**RBC (x10**^**6**^/**μl**)	**WBC (x10**^**3**^/**μl**)
Tc0	33.3	10.5	6.7	Tr0	27.2	8.4	6.1
Tc	*26.7	*8.6	6.0	Tr	*30.1	*9.5	5.7
T3	*27.7	*8.1	*9.3	Tr3	*31.9	*9.9	*7.9
T6	*27.1	*8.0	*8.5	Tr6	*31.7	9.4	*7.6
T12	*26.7	*8.8	*9.1	Tr12	*32.6	*9.7	*8.0
T24	*25.6	*7.9	7.4	Tr24	*32.0	*10.1	6.9
T48	*26.2	*6.7	7.7	Tr48	*32.2	*10.1	*7.6
T96	*26.4	*7.8	6.7	Tr96	*31.9	9.1	6.8
T8d	*27.9	**8.6	6.9	Tr8d	*35.8	*10.7	6.5

* Significative difference from time zero (T0 or Tr) throughout paired *T* test (*p* < 0.05).

The total number of leukocytes (Table 2) increased (*p* < 0.05) on T3, T6 and T12, after which, it was reduced. After blood reinfusion, a significant increase in total leukocytes (*p* < 0.05) on Tr3, Tr6, Tr12 and Tr48 was observed.

### Biochemical evaluation

Blood collection caused a decrease in TP and albumin (*p* < 0.05) (Table 3); however, these values returned to baseline 24 hours after collection. The levels of TP were below levels considered normal for the species from Tc until Tc12 (6.0–7.9 g/dL) [10], whereas albumin remained within the normal limits for the species (2.4–3.0 g/dL) [10]. Upon reinfusion, compared with Tr0, subsequent time points showed an increase in TP and albumin (*p* < 0.05).

**Table 3:** Mean of serum total protein (TP), albumin and activity of gamma glutamyltransferase (GGT) and aspartate-aminotransferase (AST) observed during the experimental time points of blood collection and reinfusion

**Time point**	**TP (g/dl)**	**albumin (g/dl)**	**GGT (U/L)**	**AST (U/L)**	**Time point**	**TP (g/dl)**	**albumin (g/dl)**	**GGT (U/L)**	**AST (U/L)**
Tc0	6.3	2.8	45	89	Tr0	5.9	2.6	42	73
Tc	*5.5	*2.5	*39	*78	Tr	*6.7	*2.9	42	79
T3	5.8	2.7	42	94	Tr3	*6.9	*2.9	*54	83
T6	5.8	*2.6	42	*98	Tr6	*6.8	*2.9	*50	83
T12	5.9	2.6	42	97	Tr12	*6.5	*2.8	*50	*86
T24	6.3	2.8	41	107	Tr24	*6.6	2.8	*51	*86
T48	6.0	2.7	41	83	Tr48	*6.3	2.7	*62	*89
T96	6.0	2.7	42	81	Tr96	*6.3	2.7	*50	76
T8d	6.0	2.7	46	77	Tr8d	*6.5	*2.9	*51	45

* Significative difference from time zero (T0 or Tr) throughout paired *T* test (*p* **<** 0.05).

GGT activity (Table 3) showed a slight reduction (*p* < 0.01) at Tc, remaining within the reference values for the species (20–52 units/L) [10]. However, after blood reinfusion, the GGT values rose, and at Tr3 and Tr48 they were 54 ± 13.2 and 62 ± 37.4, respectively, which exceeded the normal values for the species.

There was a slight decrease (*p* < 0.01) in AST serum activity at Tc and T6, while after reinfusion, there was a slight increase (*p* < 0.05) at Tr12 and Tr24.

From Tc on, there was an increase, above normal, activity of CK, but no differences between time points were observed (*p* > 0.05). From T12 on, there was a decrease in the mean CK activity, but the decrease (*p* < 0.01) was most pronounced at T48 and T96 (data not showed).

There was a reduction (*p* < 0.05) in Ca concentrations at Tc (Table 4). After reinfusion, the values for this mineral increase (*p* < 0.05) markedly from Tr. In contrast, there was a reduction (*p* < 0.05) in serum P concentration from T6 to T96 (Table 4). After reinfusion, the average P level decline (*p* < 0.05) up until Tr3, when it subsequently increased until Tr48.

**Table 4:** Mean serum concentration of creatinine, calcium (Ca) and phosphorus (P) observed during the experimental time points of blood collection and reinfusion

**Time point**	**Creatinine (g/dl)**	**Ca (mmol/l)**	**P (mmol/l)**	**Time point**	**Creatinine (g/dl)**	**Ca (mmol/l)**	**P (mmol/l)**
Tc0	1.04	8.41	9.20	Tr0	0.98	6.53	10.49
Tc	1.07	*7.15	8.96	Tr	1.03	*8.67	7.60
T3	1.04	7.50	11.85	Tr3	1.05	*8.87	*6.14
T6	0.97	7.79	*6.99	Tr6	1.05	*8.83	7.33
T12	0.94	7.73	*6.77	Tr12	1.02	*8.70	7.31
T24	1.03	7.23	7.71	Tr24	1.03	*8.60	7.20
T48	1.31	8.82	8.41	Tr48	1.06	*8.95	*6.83
T96	0.96	8.63	*7.53	Tr96	0.98	7.70	6.57
T8d	1.25	7.46	10.56	Tr8d	1.15	*8.60	*7.14

* Significative difference from time zero (T0 or Tr) throughout paired *T* test (*p* **<** 0.05).

## Discussion

The collection of 15 mL/kg of whole blood is recommended for transfusion in large animals [8,9]. In the present study, the collection and transfusion of that volume caused statistically significant changes in clinical, haematological and biochemical parameters.

After blood collection, no changes in HR were observed, whereas there was a significant decrease at reinfusion, showing that the increased volume resulting from administration of blood minimised HR in an attempt to keep the animal’s equilibrium. In this study, the HR was only evaluated after collection, not during, suggesting that withdrawal of 15 mL/kg of blood was not sufficient to activate the sympathetic response. Malikides et al. [12] has observed significant HR increases in horses during and after blood collection. In small animals and humans, decreased blood volume promotes a strong sympathetic response, leading to an increase in HR [13,14].

A significant decrease in RT was only detected in the initial time points after collection compared with the basal time point, which was probably due to decreased blood cell metabolism, but the values remained within the reference values for the species (38.5 to 39.5) [11]. Heinius et al. [15] also observed this change in a study with haemorrhagic shock in pigs.

In the sheep studied herein, there was no difference in CRT, indicating that the blood collection did not cause major damage to the animals’ circulation. According to Radostits [11], CRT reflects the circulatory status of the animal without being a high sensitivity assessment. In our study, the RR and SBP showed no differences (*p* > 0.05) between time points, despite the change in blood volume due to blood loss and reinfusion, which leads us to believe that the decline and subsequent increase in blood volume were not sufficient to cause changes that may affect these parameters.

The GV and RBC showed a decrease (*p* < 0.01) after collection but remained at levels considered normal for the species. According to Hauptman and Chaljdry [16], the GV values determined after blood loss may not be accurate, because the adaptation in the plasma/blood relationship of blood cells has not yet occurred. In spite of the significant reduction in GV and RBC from Tc to T12, there was a sharper drop at T24, suggesting that this is the time required for the stabilisation of GV and RBC. In the initial days after acute blood loss, the mobilisation of erythrocytes from storage organs such as the spleen, liver, subcutaneous tissue, great vessels and pulmonary circulation occurs as an urgent compensatory measure [17,18].

From T48, it was observed an increase in the GV and RBC, indicating that the increased production of erythrocytes is evident from 48 to 72 hours and reaches a maximum at about 7 to 8 days after the onset of bleeding [19]. In human medicine, depending on the circumstances, it is possible to collect a unit or more at appropriate intervals (every five to seven days) before elective surgery [20]. In this study, after 8 days, the GV and RBC values did not return to baseline (Tc0), and we observed that the withdrawal of 15 mL/kg of whole blood decreased the GV by 6.6%. If this is a fixed percentage, a new collection of blood after eight days would lead the animals to become anaemic, suggesting that eight days is not a sufficient interval for a new collection in sheep. Experiments with horses showed that blood withdrawal of 15 mL/kg caused GV to decline by 5% 24 hours after collection [21].

The increase in mean GV and RBC post-Tr0 are indicative of the increased supply of circulating blood constituents [22]. The reinfusion of 15 mL/kg increased GV by approximately 3%, which is similar to the increase for cows and horses undergoing homologous transfusion [8,9]. This demonstrates that autologous transfusion presents similar results to those of homologous transfusion, although it is more advantageous because it reduces the risks of adverse reactions.

The WBC initially increased and then decreased, showing that the proposed blood loss caused a slight leukocytic response, with values that are within the reference. These data suggest the autologous transfusion caused minimal complications. The early response of the organism to haemorrhagic injury is characterised by activation of the immune system and by an overwhelming inflammatory reaction [23].

Studies in human and veterinary medicine have described inflammatory responses associated with blood transfusion. In this study, reinfusion of blood caused an increase in the WBC. In an autologous transfusion model in dogs, McMichael et al. [2] used leukoreduced and nonleukoreduced erythrocyte concentrates and demonstrated that the inflammatory response was greater in animals that received the nonleukoreduced concentrate. Moreover, an in vitro study has shown a significant increase in seven markers of inflammation during storage comparing leukoreduced and nonleukoreduced erythrocyte concentrates [24].

In humans, there is an immediate 60% increase in the WBC 12 hours after transfusion, which returns to baseline within 24 hours [25]. In the sheep studied here, the WBC increased starting three hours after transfusion and decreased from the fourth day. The leukocyte response observed in this study is not linked to the presence of antibodies against the surface of erythrocytes or other cellular antigens because the animals received autologous blood. Instead, it is possibly due to storage, which promotes lysis of leukocytes along with release of cytokines and inflammatory immunomodulators such as histamine, myeloperoxidase and eosinophil cationic protein [26], which triggers an inflammatory response and consequent increase in the WBC. The reduction in WBC has been shown to lessen or eliminate the inflammatory response to blood transfusions in humans and dogs [2].

After blood collection, there was a decrease in TP and albumin, whereas there was an increase in these parameters after reinfusion. This is most likely because the average total protein is about 7% of total plasma, whereas albumin represents about 50% of plasma total protein [10,27]. Thus, the decrease in blood volume due to collection caused a decrease in TP and albumin. In a report by Kerr [28], the reduction in TP was shown to be a consequence of blood loss, and there is a tendency to lose albumin quicker than other proteins due to its small size.

Twenty-four hours after blood collection, TP and albumin values returned to similar one observed at baseline. However, Malikides et al. [12] observed the return of albumin to baseline 8 days after blood loss in horses. The rapid return to baseline levels of both TP and albumin is due to the existence of a secondary circulation of proteins (especially albumin) from the capillaries to the tissue fluids, which return to the bloodstream via the lymph [27,28].

There was an increase in TP and albumin during reinfusion (*p* < 0.05), suggesting a preservation of proteins in stored blood plasma. At the time of reinfusion, these were returned to the animal, thus increasing the values of both TP and albumin.

In this study, we evaluated the activity of a variety of enzymes in order to demonstrate changes in liver function resulting from the loss and reinfusion of whole blood. At Tc, there was a discrete decrease in GGT activity when compared to Tc0, but the values remained within the reference values for the species, suggesting that there were no hepatic injuries resulting from blood loss. After blood reinfusion, there was increased GGT activity. However, at two time points (Tr3 and Tr48), the values exceeded the reference; these were not indicative of a lesion because the values remained within normal limits at subsequent time points. Variation in AST activity was only minor, implying no major changes as it remained within the reference values for sheep (60–280 units/L) [11].

There was an increase in CK activity above the reference values from Tc until T6, which was later decreased (data not showed). The increase in activity of this enzyme may be a result of haemolysis, skeletal muscle injury or contamination of the blood sample by muscle fluid during a difficult venipuncture [27]. In this study, the need for recumbency during collection may have caused muscle damage, which increased serum levels of this enzyme. According to Kaneko et al. [10], the short half-life of CK allows for high blood levels to rapidly return to normal.

During collection and reinfusion there were no changes in creatinine and urea serum levels, indicating no kidney alteration during the both process.

Ca concentrations decreased after blood collection, but there was an increase after reinfusion. After reinfusion, the Ca level tended to increase (*p* < 0.05) markedly from Tr This illustrates that in addition to blood cells, blood reinfusion restores proteins and elements associated with them, such as Ca.

In natural cases of hypocalcaemia in bovines, there is a decrease in serum inorganic P, which can be more intense than that shown experimentally [29]. After the collection of blood from the sheep studied here, there was a reduction in P concentration, suggesting that the decrease in Ca may have contributed to this reduction. Three hours after reinfusion, P levels increased similar to the calcium levels.

## Conclusion

The blood loss caused by the collection of the 15 mL/kg volume and subsequent reinfusion of blood in sheep did not cause deleterious effects on this species. It only slightly altered the physiological, biochemical and haematological responses, indicating that the loss and haematological replacement proposed is safe and can be applied in the clinical practice of this species.

The period of eight days was not enough for the animals to fully return to baseline haematological parameters, and further research is needed to show how long it takes for the reestablishment of these parameters.

The levels of TP, albumin and Ca in the blood collected were preserved inside the blood bags, showing that autologous transfusion is a way of replacing those elements in sheep.

## Abbreviations

AST, Aspartate Aminotransferase; CK, Creatine Kinase; CRT, Capillary refill time; EDTA, Ethylenediaminetetraacetic acid; GGT, Gamma Glutamyltransferase; GV, Globular volume; HR, Heart Rate; RR, Respiratory Rate; RT, Rectal Temperature; SBP, Systolic blood pressure; TP, Time points; Tc0, Before blood collection; Tc, Ten minutes after blood collection; Tc3, Three hours after blood collection; Tc6, Six hours after blood collection; Tc12, Twelve hours after blood collection; Tc24, Twenty four hours after blood collection; Tc48, Forty eight hours after blood collection; Tc96, Ninety six hours after collection; Tc8d, Eight days after collection; Tr0, Before blood reinfusion; Tr, Ten minutes after blood reinfusion; Tr3, Three hours after blood reinfusion; Tr6, Six hours after blood reinfusion; Tr12, Twelve hours after blood reinfusion; Tr24, Twenty four hours after blood reinfusion; Tr48, Forty eight hours after blood reinfusion; Tr96, Ninety six hours after blood reinfusion; Tr8d, Eight days after reinfusion.

## Authors’ contributions

RSS, DFC, RABJ, IKFS, HSS, IOB conduct the experimental blood collection and reinfusion, collect all samples and perform the haematological analysis. AHHM make the biochemical analysis, the statistical procedures and helped with editing and revision of the manuscript. ELO designed the study and helped on the data interpretation and to write the manuscript. All authors read and approved the final manuscript.
